# Non-communicable disease comorbidities in HIV patients: diabetes, hypertension, heart disease, and obstructive sleep apnea as a neglected issue

**DOI:** 10.1038/s41598-023-39828-6

**Published:** 2023-08-05

**Authors:** Fatemeh Hadavandsiri, Maryam Shafaati, Safieh Mohammad Nejad, Mohammad Ebrahimzadeh Mousavi, Arezu Najafi, Mohammad Mirzaei, Sakineh Narouee, Samaneh Akbarpour

**Affiliations:** 1 Department of Epidemiology, School of Public Health and Safety, Shahid Beheshti University of Medical Sciences, Tehran, Iran; 2 Occupational Sleep Research Center, Baharloo Hospital, Tehran University of Medical Sciences, Tehran, Iran; 3 Research Center for Antibiotic Stewardship and Antimicrobial Resistance, Tehran University of Medical Science, Tehran, Iran; 4 Department of Microbiology, Faculty Science, Jahrom Branch, Islamic Azad University, Jahrom, Iran; 5 Department of Epidemiology and Biostatistics, School of Public Health, Tehran University of Medical, Tehran, Iran; 6 Department of Human Development and Family Sciences, University of Delaware, Newark, DE USA; 7 Hamadan Health Center, Hamadan University of Medical Sciences, Hamadan, Iran; 8 Department of Epidemiology, Kerman University Medical of Sciences, Kerman, Iran; 9 Sleep Breathing Disorders Research Center (SBDRC), Tehran University of Medical Sciences, Tehran, Iran

**Keywords:** Microbiology, Diseases, Health care, Health occupations, Medical research

## Abstract

The present study evaluates the non-communicable disease (NCD) patterns and related risk factors among people living with HIV (PLWH) in Iran. This national cross-sectional survey study was conducted on 1173 confirmed PLWHs with a mean age of 35.35 (56.82 Over 50 years old, 33.90 Under 50 years old) admitted from 15 different provinces in the country. Logistic regression was used to analyze the association of factors with having at least one NCD comorbidity. From 1173 PLWH, 225(19.18%) participants experienced at least one NCD (15.20% and 38.69% among under- and over-50-year-old patients, respectively). The prevalence of heart disease, hypertension, diabetes, and sleep apnea among all patients was 1.59%, 2.05%, 1.55%, and 10.26%, respectively. The similar prevalence for each NCD among those over 50 years was 10.11%, 15.71%, 9.01%, 25.44%, and 1.01%, 1.12%, 1.04%, and 9.23% among those under 50 years, respectively. The odds of being at risk of at least one NCD stood higher in patients over 50 years (ORadj = 2.93, 95% CI 1.96–4.37), married (ORadj = 2.48, 95% CI 1.41–4.35), divorced or widowed (ORadj = 2.78, 95% CI 1.48–5.20), and obese (ORadj = 3.82, 95% CI 2.46–5.91). According to our findings regarding the prevalence of NCDs among patients under 50 years of age, we recommend that policymakers give greater consideration to this group in the screening and care programs for NCDs since adults and the elderly are both vulnerable to the risk factors for developing NCDs.

## Introduction

According to the latest reports of the World Health Organization (WHO), there were 38.4 million people living with HIV (PLWH) globally by the end of 2021. Studies have predicted that nearly 75% of PLWH under care and treatment for HIV will be older than 50 at the end of 2030^1,2^. In Iran, model-based projections estimate that 54,850 people were HIV positive and 15,552 were under antiretroviral therapy treatment in 2021. Also, 74% of all diagnosed cases were in the age group of 20–45 years at the time of diagnosis^3^.

Although PLWH have used a large number of antiretroviral therapies (ART) in recent years, the rapid expansion of access to treatment for elderly HIV-positive patients has some challenges globally, including an increase in the comorbidity of non-communicable diseases (NCDs), such as cardiovascular disease (CVD), type 2 diabetes mellitus (T2DM), hypertension (HTN), chronic kidney disease (CKD), and cancers^4^. Consequently, this increase in NCDs’ comorbidity with HIV can be defined by improved general health, a decrease in morbidity and mortality in HIV-positive patients, and an increase in life expectancy and survival^5^.

Many studies have represented the association between aging and NCDs in PLWH ^6,7^. The prevalence of NCD risk factors among PLWH, including smoking, drug or alcohol use, comorbidity with hepatitis B or C viruses, and lower socio-economic status, is higher^8–10^. Moreover, antiretroviral therapy drugs could increase the risk of hypercholesterolemia, abdominal fat, and metabolic syndrome^11,12^. Also, in HIV-positive patients, activation in the immune system leads to the secretion of mediators and inflammatory cytokines, which results in a higher risk for atherosclerosis and coronary artery inflammation^7^. Hence, studying the comorbidity of HIV and aging has become an urgent public health concern because of the increasing number of elderly PLWH^13^.

Research in this era faced challenges due to the complex nature of NCDs in PLWH. Despite the present challenges, several studies have shown the prevalence of NCDs in PLWH in different parts of the world. However, to our knowledge, there are limited data and screening programs in the context of this emerging problem in Iran. The study of NCDs in PLWH can provide information for policymakers to better manage the overlapped epidemics of HIV and NCDs. So, the present national study aimed to address aging and NCDs, namely diabetes, hypertension, heart disease, obstructive sleep apnea co-morbidity, and related factors in PLWH.

## Methods

### Sampling method and study population

The prevalence estimation formula was used to estimate the sample size based on non-communicable disease (NCD) prevalence among PLWH^14–16^.

The study’s sample size, considering design effects and attrition, was estimated at about 1200. To allocate the appropriate sample size to each province, three provinces were randomly selected from each part of the North-East, North-West, Central, South-East, and South-West of Iran (a Total of 15 provinces), and the total sample size was divided based on proportion to the size of diagnosed patients. Next, a list of VCTs was prepared separately for each of the 15 provinces; then, two VCTs in each of these provinces were randomly selected for data collection. Finally, in each of the VCTs, convenience sampling was used to choose participants.

### Collecting data

For data collection, trained interviewers with experience working on PWLH using standard questionnaires collected data from adult patients aged ≥ 18 diagnosed at least 3 months ago. Verbal informed consent was also obtained from patients all over Iran between April 2021 and March 2022.

Data collection consisted of two parts: a physical examination and a questionnaire. The physical examination consisted of height and weight measurements. A demographic questionnaire and the Berlin Obstructive Sleep Apnea Questionnaire (BQ) were used for data collection. This scale has ten items assessing obstructive sleep apnea risk factors, including snoring, excessive daytime sleepiness and fatigue, obesity, and high blood pressure. A positive score on more than one subscale of the three shows a high risk of developing sleep apnea^17^. The validity and reliability of this tool have been confirmed with a Cronbach’s alpha coefficient of 0.9^18^ for the original version and 0.90 for the Persian version^19^.

Self-reported NCD diseases were considered by asking questions about diagnosed diseases by physicians or using related medications in the included questionnaire. Each NCD, heart disease, hypertension, diabetes, was defined as patients who had diseases diagnosed by a physician or were on medications. Medical information and disease-related data—including duration of HIV diagnosis, duration of ART, ways of HIV transmission, hepatitis C virus, hepatitis B virus, and tuberculosis (TB) comorbidity, the last CD4 count, and the last viral load—extracted from the patient's electronic health record available in VCTs.

### Ethical consideration

Ethical approval was received for this study from the Tehran University of Medical Sciences (TUMS) that all research was performed in accordance with relevant guidelines and regulations (ethics approval number: IR.TUMS.FNM.REC.1399.066), and all processes were performed in accordance with the Declaration of Helsinki.

Every participant provided verbal informed consent, according to patients with HIV/AIDS research approved by the Tehran University of Medical Sciences Ethics Committee under the code IR.TUMS.FNM.REC.1399.066,

### Statistical analysis

Although data were gathered for 1200 PLWH cases, 27 patients had missing data on at least one of the NCDs that were excluded from the study, and data analysis was performed on 1173 patients with complete NCD data. Other socio-demographic and clinical characteristics with missing values (3.01% of the whole dataset) were imputed by performing a single imputation and regression model.

All analyses were carried out using complex survey methods to regulate cluster sampling and sample weight. Based on the age and sex categories of the 15-year-old national Iranian PLWH population in 2021, the data were weighted. The weighted mean with standard error (SE) and weighted percent were used to first report the characteristics of the individuals. In the complex survey approach, a t-test analysis (*p* < 0.05) was used to examine differences in age, body mass index (BMI), duration of HIV diagnosis, CD4 count, viral load, and ART duration between the under-50 and over-50-year-old groups and a chi-square test was used for other qualitative variables. The normality of continuous data was checked by using a histogram.

Logistic regression was used to investigate the relationship between baseline variables and having at least one NCD comorbidity with HIV in total patients. For exploring associated factors, a univariable analysis was performed, and variables with a *p*-value < 0.1 were entered in the multiple regression model as the final model. All analyses were performed using STATA (17 version) for complex survey analysis, and *p*-values < 0.05 were considered statistically significant.

## Results

The present research enrolled 1173 PLWH in the analysis, with a mean age of 35.35 years (SE: 0.06). Of these, 974 (83.03%) and 199 (19.97%) cases were aged under 50 and over 50, respectively. The mean age of participants under 50 and over 50 was 33.90 (SE:0.07) and 56.82 (SE: 0.22) years, respectively. The CD4 count and median of the duration of HIV diagnosis (month) were higher in people under 50, and in terms of NCD prevalence, there is a significant difference between the two age groups (*p* < 0.001) (Table 1).

**Table 1:** Socio-demographic and clinical characteristics between the two groups defined by age in patients living with HIV.

Variables	Total (n = 1173)	Under 50 years (n = 974)	Over 50 years (n = 199)	*P*-value
	Mean (SE)	Mean (SE)	Mean (SE)	
Age (years)	35.35 (.06)	33.90 (0.07)	56.82 (0.22)	*P* < 0001
BMI	23.35 (0.18)	23.17 (0.19)	26.04 (0.34)	*P* < 0001
CD4 count (cells/mm_2_)	426.99 (18.06)	433.11 (19.22)	336.98 (24.04)	0.02
Viral load	135,611.1 (20,324.31)	131,018.5 (21,003.13)	203,221.8 (82,043.81)	0.59
Duration of HIV diagnosed, month (median, IQR)	84.5 (92.16)	87.16 (89.3)	66.93 (88.04)	0.02
Duration of ART, months (Med, IQR)	55 (60)	54 (57)	60 (70)	0.51

Variables	Total (n = 1173)	Under 50 years (n = 974)	Over 50 years (n = 199)	*P*-value
	Frequency (%)	Frequency (%)	Frequency (%)	
Sex				
Male	709 (81.28)	575 (81.67)	134 (75.51)	0.02
Female	464 (18.72)	399 (18.33)	65 (24.49)	
Marital status				
Single	262 (43.63)	245 (45.96)	17 (9.37)	*P* < 0.001
Married	606 (41.34)	494 (40.16)	112 (58.68)	
Divorced or widowed	305 (15.03)	235 (13.88)	70 (31.94)	
Education				
Under diploma	745 (50.52)	587 (48.66)	158 (77.98)	*P* < 0.001
Diploma	339 (37.26)	306 (38.60)	33 (17.58)	
Upper diploma	89 (12.22)	81 (12.74)	8 (4.44)	
BMI category (kg/m^2)^				
< 25	707 (66.03)	624 (67.75)	83 (40.61)	*P* < 0.001
25–30	298 (21.27)	221 (20.01)	77 (39.77)	
> = 30	168 (12.70)	129 (12.24)	39 (19.62)	
Employment status				
Employed	577 (57.12)	467 (56.87)	110 (60.67)	0.05
Unemployed	596 (42.88)	507 (43.13)	89 (39.33)	
Route of transmission				
Sexual contact	222 (35.66)	194 (37.02)	28 (15.74)	0.15
Injection drug use	398 (34.56)	328 (34.24)	70 (39.30)	
Unknown^1^	553 (29.77)	452 (28.74)	101(44.97)	

^1^Transmission from mother to child, High Risk Spouse, Infected Spouse, Occupational Transmission were defined as unknown.

As shown in Table 2, the prevalence of HCV, HBV, and TB comorbidity in PLWH under 50 was 13.86%, 2.33%, and 5.38%, and in PLWH over 50, it was 19.69%, 3.38%, and 7.13%, respectively.

**Table 2:** Diseases comorbidities by age in patients living with HIV.

Variables	Total (n = 1173)	Under 50 years (n = 974)	Over 50 years (n = 199)	*P*-value
	Frequency (%)	Frequency (%)	Frequency (%)	
Communicable comorbidity				
HCV	188 (14.23)	153 (13.86)	35 (19.69)	0.51
HBV	34 (2.40)	28 (2.33)	6 (3.38)	0.91
TB	83 (5.49)	70 (5.38)	13 (7.13)	0.74
Non-communicable comorbidity				
Heart disease	41 (1.59)	21 (1.01)	20 (10.11)	*P* < 0.001
Lung disease	20 (1.61)	17 (1.60)	3 (1.71)	0.81
Hypertension	54 (2.05)	23 (1.12)	31 (15.71)	*P* < 0.001
Diabetes	37 (1.55)	19 (1.04)	18 (9.01)	*P* < 0.001
Obstructive sleep apnea	154 (10.26)	103 (9.23)	51 (25.44)	*P* < 0.001
Number of Non-Communicable comorbidities				
0 comorbidity	948 (86.13)	826 (87.84)	122 (61.04)	*P* < 0.001
1 comorbidity	165 (11.39)	121(10.63)	44 (22.50)	
2 comorbidities	44 (1.97)	21 (1.32)	23 (11.49)	
3 and more comorbidities	16 (0.51)	6 (0.21)	10 (4.97)	
At least one comorbidities (one and more)	225 (19.18)	148 (15.20)	77 (38.69)	*P* < 0.001

HCV, hepatitis C virus; HBV, hepatitis B virus; TB, Tuberculosis.

About 225 (19.18%) participants experienced at least one NCD (15.20% and 38.69% among under- and over-50-year-old patients, respectively). The prevalence of heart disease, hypertension, diabetes, and sleep apnea among all patients was 1.59%, 2.05%, 1.55%, and 10.26%, respectively. The similar prevalence for each NCD among those over 50 years was 10.11%, 15.71%, 9.01%, 25.44%, and 1.01%, 1.12%, 1.04%, and 9.23% among those under 50 years, respectively.

The prevalence of one, two, or three and more non-communicable comorbidities in patients under 50 was 10.63%, 1.32%, and 0.21%, respectively, and patients over 50, it was 22.50%, 11.49%, and 4.97%, respectively.

**Figure 1:**
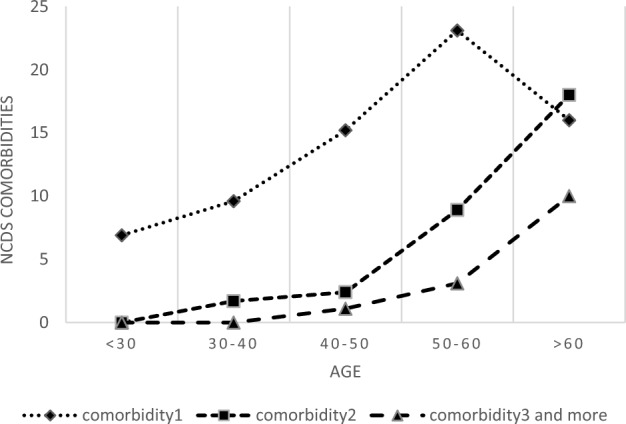
NCDs comorbidity with HIV by age.

As Fig. 1 shows, the prevalence of comorbidities increases with age. The peak of one comorbidity occurred in the age group 50–60, and two and three comorbidities occurred in the age group over 60.

Multiple logistic regression was used to address factors associated with having at least one NCD comorbidity. The results of multivariate logistic regression showed that several factors were positively associated with having NCD in PLWH. Age over 50 years (ORadj = 2.93, 95% CI 1.96–4.37), married participants (ORadj = 2.48, 95% CI 1.41–4.35), divorced or widowed (ORadj = 2.78, 95% CI 1.48–5.20), and BMI ≥ 30 (ORadj = 3.82, 95% CI 2.46–5.91) were associated with having at least one NCD comorbidity in PLWH. Nevertheless, patients with an education level diploma had lower odds of at least one non-communicable comorbidity than those under diploma counterparts (ORadj = 0.64, 95% CI 0.41–0.97) (Table 3).

**Table 3:** Factors associated with having at least one non-Communicable comorbidity in the logistic regression model.

Outcome	ORadj*	95% CI	*P*-value
Age			
Under 50 years	Ref		
Over 50 years	2.93	1.96–4.37	*P* < 0.001
CD4 count (cells/mm^2^)	0.99	0.99–1.0005	0.947
Duration of HIV diagnosed, month (median, IQR)	0.99	0.99–1.001	0.342
Sex			
Male	Ref		
Female	0.74	0.466–1.17	0.203
Marital status			
Single	Ref		
Married	2.48	1.41–4.35	0.002
Divorced or widowed	2.78	1.48–5.20	0.001
Education			
Under diploma	Ref		
Diploma	0.64	0.41–0.97	0.040
Upper diploma	0.67	0.30–1.51	0.347
BMI category (kg/m^2^)			
< 25	Ref		
25–30	1.09	0.71–1.66	0.676
> = 30	3.82	2.46–5.91	0.000
Employment status			
Unemployed	Ref		
Employed	0.97	0.62–1.50	0.909

*ORadj: Adjusted odds ratio.

## Discussion

Our findings demonstrated that the chance of having at least one noncommunicable disease was higher in patients over 50 years old. Age and obesity were significant risk factors for NCDs in those over 50, while HIV transmission through sexual contact, obesity, level of education, and age were significant risk factors in patients under 50. This study was consistent with other studies^20^. For those who have HIV infection, the comorbidity of NCDs is becoming a greater burden. However, information on the extent of HIV-NCD comorbidity and the factors that contribute to it in Iran is scarce. With the improvement in HIV-infected individuals’ survival, the probability of other diseases is increasing, like in the general population^21,22^. According to reports from the World Health Organization, ART may affect and progress NCDs^10^. By increasing NCD risk factors in low-resource settings, the prevalence of NCDs increases and shifts from the affluent to the less affluent population^23^. One of the main causes of NCDs among PLWH is aging, which is called accelerated aging and leads to NCDs affecting PLWH decades earlier than people without HIV^24^. The objective of this study was to determine the effect of aging and non-communicable comorbidities in PLWH.

Obesity is known as a risk factor for NCDs and diabetes and is a behavioral risk factor and consequence of lifestyle^25^. The increase in overweight and obesity in low-resource-setting countries is greater than in high-resource-setting countries^26^. Also, obesity in men and women is associated with higher socioeconomic status, whereas in high-income countries it is related to poverty^27^. Studies show that as individual’s age, their risk of NCDs considerably increases. In the current study, age and obesity were found to be predictors of HIV-NCD comorbidity. Numerous studies have shown that the incidence of NCDs increases with age. Investigations, for instance, showed that impairments in glucose tolerance were more prevalent in people who were older^28^.

Some investigators agree with the finding that PLWH on ART have a lower BMI and a lower prevalence of obesity and overweight than their counterparts^29^. It could be attributed to the later initiation of ART^30^. Weight loss and wasting are common symptoms of HIV-related illness. However, it is well-established that antiretroviral therapy (ART) can reverse HIV-related weight loss and wasting^31^. The results of this comprehensive study on NCD comorbidities in PLWH showed that the prevalence of obesity in patients over 50 years was 19.60%.

PLWH on ART are at higher risk of NCDs at older ages in the present study. Older age increases the odds of NCD comorbidity by 5% and 8% in patients under 50 and over 50, respectively. The patients younger than 30 years faced one NCD. As patients get older, they encounter more than one NCD. The peak of multi-morbidity in patients occurred at ages 60 and older. Although communicable and lung disease comorbidities were not significant, the prevalence of all non-communicable comorbidities was higher in patients over 50 years. The results of the Oni et al. study show a higher prevalence of multi-morbidity in younger ages (18–35 years) for women and, by contrast, in older ages (46–55 years) for men, whereas after adjusting for age, comorbidity in younger ages was more prevalent^32^. The suggested reason for this was previously mentioned as being premature aging^33^. In contrast, the study conducted by Zhang et al. found that the elderly population in China has a high prevalence of multi-morbidity^34^. Antiretroviral therapy (ART) has decreased virological failure, but the results of a cohort study on the initiation of ART show those patients who start ART at 50 years of age or older are at higher risk for death than those who start ART at younger ages^35^.

In our study, there was no significant association between men and women in disease comorbidities with HIV. In a study in South Africa, women were at higher risk for obesity than men^36^. Oni et al. confirm the significant difference between men and women in multi-morbidity patterns^32^. In a systematic review, results confirm the prior finding about higher odds of comorbidities in females versus^37^. This finding was in line with the 2018 Academy of Medical Science report about multi-morbidity^38^. A higher rate of comorbidity in females could be explained by more self-reporting^39^, higher life expectancy^40^ and adherence to healthcare providers’ advice, and frequency of visits^41^.

A higher prevalence of NCDs in PLWH than in the past could be due to a combination of several factors, like aging, more affection for PLWH by traditional NCD risk factors, and the effects of human viruses and ART side effects^42^. ART effects on lifespans led PLWH to be exposed to NCD risk factors as much as normal people^7,16^. The rapid change in demographic characteristics of this population and the epidemiological transition enhanced by unhealthy behaviors such as an unhealthy diet and lower physical activity could increase the chance of NCDs^43^. Hypertension is one of the main cardiovascular disease (CVD) risk factors^44^. Our results confirm the previous finding that hypertension is one of the most common comorbidities among PLWH. In this study, 16.08%, 9.05%, and 6.53% of PLWH aged 50 and older were impressed by hypertension, diabetes, and TB, respectively. A study that examined the comorbidity pattern of HIV, TB, and NCDs reported that the prevalence of hypertension in individuals 50 years of age and older was 77%, and it was the most common comorbidity in HIV patients on ATR^14^. Also, Kaluvu et al., in a systematic review, cited hypertension as the most frequent (23.3%) comorbidity with HIV. Diabetes, TB, and HIV (26.6%) and hypertension, diabetes, and HIV were the most frequent triples (63%)^37^. The results of a meta-analysis conducted in low- and high-income countries showed that the prevalence of HIV comorbidity due to hypertension and obesity was 21.2% and 7.8%, respectively^16^.

In the present study, the prevalence of HCV and HBV in PLWH older than 50 was higher than in their counterparts younger than 50, but the difference was not statistically significant. In a study in India, the prevalence of HBV and HCV among HIV-positive participants who injected drugs was 9.7% and 53.7%, respectively^45^. The prevalence of HCV and HIV comorbidity in homeless men was 5.76% in a study in Iran^46^. The comorbidity of HIV with viral hepatitis because of some complications such as liver problems, renal disorders, immunological and hematological problems, and CVD must be considered^47^.

To the best of our knowledge, this study is the first such effort to investigate the current health service landscape for populations in Iran that are co-morbid with HIV and NCDs.

The finding of the present study demonstrates that obstructive sleep apnea affects 25.63% of PLWH aged 50 and older, which could be due to the higher prevalence of obesity in older ages. In a study by Njoh et al., the prevalence of HIV infection in patients with sleep apnea was higher than in those without sleep apnea syndrome. This study reveals a strong association between HIV and obstructive sleep apnea^48^, which is the opposite of our study’s finding. It may be due to different study designs (case–control study vs. cross-sectional study).

Although there is little information on integrated HIV care and NCD services, shreds of evidence show it could be beneficial for PLWH^49^. So that most of the risk factors are behavioral, and the majority of them potentially require intervention^50^. The NCDs as emerging problems among PLWH must be confronted and leveraged through HIV health care investments to enhance well-being and reduce mortality among PLWH^51^. Thereby, early detection of risk factors of NCDs as preventive behaviors would reduce the costs imposed, morbidity, and mortality.

This study was conducted on the national data of PLWH. These data were used to determine the effect of aging on the comorbidity of HIV with NCDs for the first time in the country, which is the most important strength of the study. Also, we examined obstructive sleep apnea in addition to NCDs.

Despite the promising results of this study, there are some limitations that should be acknowledged. Due to the coincidence of the COVID-19 era, we had to use convenience sampling based on the regular visits of HIV patients to VCTs to get their own antiretroviral regimens. We try to use weighted analysis to manage this issue. On the other hand, the measurement of non-communicable diseases here was stated through self-report and was not measured accurately for each patient, which led to an underestimation of the number of samples.

## Conclusion

In general, the study found that patients over 50 had a considerable amount of comorbidity. Being older and having a BMI over 25 increased the chance of developing NCDs in people living with HIV. Targeted NCD prevalence screening and caring, addressing modifiable risk factors, and providing integrated care are all advised in order to improve the quality of life for comorbid patients.

It is essential to make investments in improved NCD management. Making sure individuals who require it have access to palliative care is a part of the detection, screening, and treatment of NCDs. We focused on the comorbidity of NCDs in PLWH because it is a growing problem in the context of HIV-positive patients. Policymakers should take into account these issues, adopt coordinated management between HIV and chronic disease care, and promote NCD screening programs for PLWH due to the impact of aging on the growing NCDs and the rising population of PLWH aged under and beyond 50.

This national study, which was conducted in Iran, revealed that there is typically no screening policy for non-communicable diseases in PLWH. However, we also emphasized the significance of NCD prevalence in patients under the age of 50 compared to the general population; this group should not be ignored. In order to get medical professionals and health policymakers to pay more attention to the spread of non-communicable diseases in PLWH, we recommended that HIV patients be checked for comorbidity with non-communicable diseases.

## Data Availability

The datasets generated and/or analysed during the current study are not publicly available due to[REASON WHY DATA ARE NOT PUBLIC] but are available from the corresponding author on reasonable request.
